# Optimizing Surgical Choice in Mild and Moderate OSA: Anterior Palatoplasty vs. Radiofrequency Uvulopalatoplasty

**DOI:** 10.3390/life16040687

**Published:** 2026-04-18

**Authors:** Ionut Tanase, Mircea-Sorin Ciolofan, Codrut-Caius Sarafoleanu, Mihaela Cristina Neagu, Florentina-Carmen Badea, Carmen Aurelia Mogoantă

**Affiliations:** 1 Department of Otorhinolaryngology, Carol Davila University of Medicine and Pharmacy, 050474 Bucharest, Romania; ionut.tanase@umfcd.ro (I.T.); codrut.sarafoleanu@umfcd.ro (C.-C.S.); 2 Department of Otorhinolaryngology, University of Medicine and Pharmacy of Craiova, 200349 Craiova, Romania; carmen.mogoanta@umfcv.ro; 3 ENT Department, Medicover Hospital, 020331 Bucharest, Romania; carmeen.badea@gmail.com

**Keywords:** anterior palatoplasty, radiofrequency-assisted uvulopalatoplasty, obstructive sleep apnea, sleep surgery, polysomnography

## Abstract

Background: Surgical palatal techniques are established alternatives to continuous positive airway pressure (CPAP) in selective patients with obstructive sleep apnea (OSA) with retropalatal airway collapse. Anterior palatoplasty (AP) stiffens and advances the soft palate, whereas radiofrequency-assisted uvulopalatoplasty (RF-UPP) uses thermal ablation to reduce palatal tissue. This study aimed to compare the 6-month efficacy and morbidity of AP vs. RF-UPP in treating mild-to-moderate OSA. Materials and Methods: We conducted a single-center retrospective cohort study (March 2023–March 2025) of 86 adults (mean age ~42 years; 69.8% male) with mild-moderate OSA (apnea–hypopnea index [AHI] 5–30) due to palatal obstruction; 43 patients underwent AP and 43 patients underwent RF-UPP. Polysomnographic AHI, Epworth sleepiness scale (ESS), snoring severity (0–10 visual analog scale, VAS) and sleep-related quality of life (functional outcomes of sleep questionnaire, FOSQ) were analyzed at baseline and 6 months postoperatively. Postoperative pain (0–10 VAS), recovery time, and bleeding events were also assessed. Results: Baseline characteristics were similar between groups (AHI ~22 vs. 21 events/h; ESS ~11 vs. 10; snoring VAS ~8.4 vs. 8.2 in AP vs. RF-UPP, all *p* > 0.1). At 6 months, the AP group achieved a greater mean AHI reduction than the RF-UPP group (−13.5 ± 7.5 vs. −8.0 ± 7.2, *p* < 0.001), with post-treatment AHI averaging 8.5 ± 6.0 vs. 13.2 ± 6.5 events/h (AP vs. RF-UPP). AP yielded a higher surgical success rate (34/43 (79.1%) vs. 23/43 (53.5%), *p* = 0.012), meeting the criteria of ≥50% AHI reduction to <15; *p* = 0.01. Subjective outcomes improved in both groups, but AP showed greater mean reductions in ESS (−5.5 vs. −3.1 points, *p* = 0.001) and snoring VAS (−5.7 vs. −3.1, *p* = 0.002). The improvements in ESS, snoring VAS, and FOSQ scores were observed in both groups, with significantly greater gains after AP. Postoperative pain and time to resumption of normal diet were higher in the AP group. No major complications occurred in either group. Conclusions: Anterior palatoplasty demonstrated superior efficacy to RF-UPP in mild-moderate OSA at the expense of increased postoperative pain and a longer recovery period. AP may offer a greater therapeutic benefit in appropriately selected patients with palatal obstruction.

## 1. Introduction

Obstructive sleep apnea (OSA) is a prevalent condition characterized by recurrent upper airway collapse during sleep, leading to intermittent hypoxemia and sleep fragmentation. It affects an estimated of 5–15% of adults, and it is associated with significant cardiovascular morbidity [1]. First-line therapy for OSA is usually CPAP, which pneumatically splints the airway open [2]. However, many patients with mild or moderate OSA find CPAP burdensome and seek alternative treatments. In selected patients with anatomical anteroposterior narrowing at the retropalatal level, surgical intervention on the soft palate can enlarge the airway and reduce collapsibility [3,4].

OSA severity is classified by the American Academy of Sleep Medicine (AASM) and the International Classification of Sleep Disorders, according to the apnea–hypopnea index (AHI): mild OSA is defined as AHI 5–14 events/h, moderate OSA as AHI 15–29 events/h, and severe OSA as AHI greater than 30 events/h [5]. Each tier carries progressively greater cardiovascular, metabolic, and neurocognitive risk, and treatment decisions are guided primarily by severity in conjunction with symptom burden and comorbidity profile. It should be noted that while no formal guideline defines a severity category beyond these three tiers, the clinical literature recognizes patients with very high AHI, profound nocturnal hypoxemia, and complex multilevel obstruction whose disease course may be life-threatening; such patients represent a distinct phenotype, typically requiring multilevel or combined surgical device approaches and fall outside the scope of the present study.

The management of OSA encompasses a broad therapeutic spectrum stratified by disease severity, patient anatomy, and individual tolerance, as summarized in Table 1. Conservative measures—including positional therapy, weight reduction, and avoidance of sedatives and alcohol—represent first-line intervention for mild OSA. Continuous positive airway pressure (CPAP) therapy remains the gold standard for moderate and severe disease with robust evidence for the AHI reduction, improvement in daytime sleepiness, and cardiovascular risk mitigation [2]. However, long-term CPAP adherence remains a significant clinical challenge, with the studies reporting abandonment rates exceeding 50% at one year, particularly among patients with mild-to-moderate OSA [2]. Oral appliance therapy, endorsed by the AASM and the American Academy of Dental Sleep Medicine as a first-line alternative for mild and moderate OSA and for patients intolerant to CPAP, achieves meaningful AHI reduction through mandibular advancement [6]. Surgical options encompassing nasal surgery, palatal procedures, tongue base reduction, transoral robotic surgery, maxillomandibular advancement, and hypoglossal nerve stimulation—are increasingly recognized as legitimate primary or alternative therapies when patient anatomy, preference, or CPAP intolerance are present [7].

The current approach to OSA treatment is largely based on two key reference documents: the AASM 2019 guideline for positive airway pressure (PAP) therapy and the ERS 2021 guideline for non-CPAP treatment options [2,8].

Although some areas have been updated more recently, such as AASM 2025 guidance for hospitalized patients with OSA, there is still no single guideline that brings together all treatment options into a unified framework [9].

The ERS 2021 guidelines have been further supported by the World Sleep Society endorsement in 2024 [10]. More recently, the 2023 International Consensus Statement on OSA has provided a detailed multidisciplinary overview, although it represents expert consensus rather than a formal guideline [11].

**Table 1:** Therapeutic options for obstructive sleep apnea stratified by severity and treatment indications.

Category	Modality	Indication by Severity
Conservative/behavioral	Weight reduction, positional therapy, sleep hygiene, myofunctional therapy	First-line adjunct for all severities
Positive airway pressure	CPAP/auto-CPAP/BiPAP	Gold standard first-line for moderate-severe OSA
Oral appliance therapy	Mandibular advancement devices	First-line alternative for mild-moderate OSA; second-line alternative for severe OSA when CPAP fails
Nasal surgery	Septoplasty, turbinate reduction, nasal valve repair	Adjunct in all severities; improves CPAP compliance and reduces titration pressures
Palatal surgery—minimally invasive	RF-UPP, palatal implants	Mild OSA with retropalatal collapse; limited efficacy as a stand-alone treatment in moderate-severe OSA
Palatal surgery—reconstructive	AP, ESP, BRP, lateral pharyngoplasty	Mild-moderate OSA; as part of multilevel approach in severe cases
Tongue base/hypopharyngeal surgery	Tongue base reduction, TORS, genioglossus advancement, hyoid suspension	Moderate-severe OSA; typically in multilevel approach
Hypoglossal nerve stimulation	Upper airway stimulation	Moderate-severe OSA
Maxillofacial surgery	Maxillomandibular advancement	Moderate-severe OSA with craniofacial abnormalities or multilevel collapse; after failed soft tissue surgery
Bariatric surgery	Sleeve, bypass	BMI over 35 with OSA
Tracheostomy		Last resort for life-threatening refractory severe OSA

References: [2,8,10,11].

It is important to recognize that isolated nasal or palatal surgery represents only one treatment pathway within the broader OSA management algorithm, applicable to a phenotypically defined patient subgroup with a site-specific obstruction. Significant multilevel pharyngeal collapse, craniofacial skeletal deficiency, or morbid obesity typically require alternative or combined therapeutic approaches—including multilevel surgery or continued PAP therapy. The present study focuses exclusively on two palatal techniques in patients with confirmed isolated retropalatal obstructing in mild and moderate disease severity.

Accurate patient selection for palatal surgery requires a structured preoperative evaluation combining clinical and paraclinical examination. Objective nasal patency is assessed by active anterior rhinomanometry, which quantifies total nasal airway resistance. In the present study, rhinomanometry was performed in patients who cleared initial clinical screening and demonstrated no clinically obvious nasal obstruction in order to objectify nasal air patency prior to surgical planning. Drug-induced endoscopy (DISE) has emerged as the essential dynamic tool for OSA surgical planning, allowing direct visualization of the site, degree, and configuration of pharyngeal collapse under pharmacologically induced sleep using VOTE classification—assessing velum, pharyngeal walls, tongue base, and epiglottis by degree of obstruction (none, partial, complete) and collapse pattern (anteroposterior, lateral, concentric)—to characterize each obstruction level with precision [12]. In our study, DISE was performed only on patients that cleared initial clinical selection—that is, only in patients who had already demonstrated predominantly retropalatal collapse and who met the including criteria.

Anterior palatoplasty (AP) was developed as a less invasive palatal surgery to treat snoring and mild-moderate OSA [13,14]. In AP, a strip of soft palate mucosa is removed, and the palatal tissue is advanced and sutured forward, stiffening and shortening the palate [3]. This technique increases the retropalatal airway by inducing fibrotic scarring that prevents the collapse [4,15,16]. AP can be performed alone or in conjunction with tonsillectomy, and it has shown significant reductions in the AHI (from ~16 to 7 on average) and snoring intensity, with surgical success rate around 72.5% in mild-moderate OSA [4]. Notably, comparative studies indicate that AP yields greater improvements in the AHI, ESS, and snoring severity than older techniques like the uvulopalatal flap or modified UPPP [15]. It has also been associated with less postoperative pain than traditional flap or resection techniques in some series [17].

Radiofrequency uvulopalatoplasty is a minimally invasive approach introduced in the late 1990s as an office-based treatment for snoring and mild OSA [18]. RF-UPP uses low-temperature radiofrequency energy delivered via electrode probes to create submucosal thermal lesions in the soft palate and partial resection of uvula, leading to localized tissue fibrosis and shrinkage upon healing. The goal is to stiffen the palate without extensive resection. Radiofrequency palatal procedures are generally well tolerated with a few complications and substantially less postoperative pain compared to UPPP [19,20,21]. However, multiple treatment sessions are often required to achieve optimal results, and efficacy in reducing the AHI has been variable [22]. Prior studies have shown that radiofrequency palatal surgery can significantly improve subjective snoring and daytime sleepiness [22]. For example, Tschopp et al. reported that RF-UPP combined with tonsillectomy reduces ESS from 8.4 to 4.1 and snoring VAS from 7.9 to 3.3 on average at 3 months [23]. Objective AHI reductions with RF are usually modest (often on the order of 20–40% in mild-to-moderate OSA) and may be less pronounced than those reported for surgical palatoplasty techniques in some series [4,24]

Given the range of options for palatal OSA surgery, there is a need for comparative effectiveness data to guide treatment selection. To date, few studies have directly compared AP to RF-UPP surgery in obstructive sleep apnea. We hypothesized that AP, by structurally enlarging the velopharyngeal airway, would result in greater AHI reduction and symptom improvement than RF-UPP in patients with mild-to-moderate OSA, albeit with a trade-off of higher postoperative morbidity. To test this, we reviewed the outcomes of patients who underwent either AP or RF-UPP over a 2-year period. The primary objective was to compare the change in the AHI at 6 months postoperatively between the two procedures. Secondary objectives included the comparisons of ESS, snoring VAS, and the quality of life scores, as well as the evaluation of postoperative pain, recovery time, and any bleeding complications. This study aims to guide surgical choice in OSA by providing comparative data on the palatal techniques, highlighting the efficacy and tolerability of anterior palatoplasty versus radiofrequency in a real-world cohort.

## 2. Materials and Methods

### 2.1. Study Design and Patients

We performed a retrospective cohort study of adult OSA patients who underwent palatal surgery at a tertiary hospital between March 2023 and March 2025. The study was approved by the institutional ethics committee (approval no. 28689/16.10.2025). Informed consent was obtained from all subjects involved in the study.

All patients underwent baseline overnight PSG within 2–3 months prior to surgery. PSG was scored according to standard AASM criteria [25]. The interval between the diagnostic PSG and surgical intervention did not exceed 3 months in any patient, ensuring that baseline respiratory parameters accurately reflected the patient’s status at the time of the surgery. PSG was performed using the Alice system (Philips Respironics, Murrysville, PA USA), with data acquisition conducted using the Sleepwear G3 software version 3.9.5 (Respironics Inc., Murrysville, PA, USA ).

All patients underwent the standardized preoperative evaluation protocol prior to surgical planning. Clinical evaluation and flexible fibreoptic nasopharyngolaryngoscopy was performed in all patients to dynamically assess airway patency. In patients who cleared initial clinical screening and demonstrated no clinically obvious nasal obstruction, rhinomanometry was performed to objectify total airway nasal resistance (NAR). A total NAR exceeding 0.30 Pa/cm^3^/s was considered indicative of clinically significant nasal obstruction, and such patients were excluded. DISE was subsequently performed on all patients who met clinical eligibility criteria using propofol-based sedation. Pharyngeal collapse was classified using the validated VOTE system [12,26].

Patients in this cohort presented with the clinical symptom profile characteristic of mild-to-moderate OSA. The predominant presenting complaints included habitual snoring reported by partners, witnessed apnoeic episode, excessive daytime sleepiness, impaired concentration, reduced work capacity, and non-restorative sleep. These symptoms were formally quantified using three validated instruments: the Epworth sleepiness scale, the snoring severity visual analog scale, and functional outcomes of sleep questionnaire (FOSQ), as reported in Table 2.

OSA symptom burden broadly increases with disease severity; however, the relationship between subjective symptoms and the AHI remains heterogenous. Excessive daytime sleepiness, while more prevalent in severe cases, remains highly variable, though the relationship with the AHI is non-linear, and individual variability is substantial; a clinically relevant subset of patients demonstrate normal ESS values despite substantial event burden [27]. Neurocognitive symptoms, including impaired attention, memory deficits, and reduced executive function, are consistently documented across all severity tiers, and are understood to result from intermittent hypoxia and sleep fragmentation [28].

OSA is associated with impaired daytime functioning and reduced health-related quality of life, with symptoms extending beyond respiratory events, affecting daily performance, alertness, and social functioning [29]. The functional outcomes of sleep questionnaire is particularly useful in this context because it measures sleepiness-related difficulties and domains such as activity level, vigilance, productivity, social outcomes, and intimacy, thereby reflecting how OSA symptoms translate into everyday functional burden [30]. In patients with mild-to-moderate OSA, these impairments are often present but tend to be more selective and less disabling overall, affecting concentration, work efficacy, and energy rather than causing uniformly severe dysfunctions across all domains [31].

Routine cephalometry or cone beam computed tomography was not performed as part of the standardized preoperative protocol. Craniofacial and skeletal abnormalities were assessed on clinical examination, including jaw position, dental occlusion, and facial symmetry. Patients presenting with clinically evident skeletal malformation were excluded at the initial triage and redirected to appropriate management pathways. As no patient in the final cohort exhibited clinical signs of significant skeletal deformities, advanced imaging was not pursued. This represents a study limitation, as subtle skeletal contributions to retropalatal narrowing cannot be entirely excluded.

The Mallampati classification is used as part of the routine clinical examination in all patients, particularly in the context of pre-anesthetic airway assessment. However, it was not used as a primary determinant for surgical planning. The Mallampati score represents a static, awake assessment of the tongue–palate size ratio, which does not capture the dynamic pharyngeal collapse patterns occurring during sleep [32].

Studies have demonstrated only a slight concordance between the Mallampati classification and the DISE findings [33], while DISE has been shown to alter surgical plans in approximately 50% of cases compared to clinical examination alone [34]. Accordingly, the site, degree, and configuration of pharyngeal collapse were determined by DISE using the validated VOTE classification, which provides dynamic assessment of anteroposterior, lateral, and concentric collapse pattern at each anatomic level—the information that static clinical scores cannot capture. Palatal morphology, including lengths, redundancy, and thickness, was assessed qualitatively by the operation surgeon during clinical examination and was confirmed during DISE.

A formal a priori sample size calculation was not performed, given the retrospective design; however, a post hoc power analysis based on the observed mean between-group differences in the AHI reduction (5.5 events/h, pooled SD 7.35, Cohen’s d = 0.75) confirmed that the final cohort provided 92.9% statistical power to detect this difference at a two-sided alpha of 0.05 (independent samples *t*-test).

### 2.2. Inclusion and Exclusion Criteria

Inclusion Criterion: Age ≥ 18 years. Confirmed diagnosis of OSA with baseline apnea–hypopnea index between 5 and 30 events/h (mild or moderate OSA). Isolated primary site of airway collapse at the soft palate level evaluated initially by fibreoptic endoscopy and confirmed by DISE underwent either anterior palatoplasty or radiofrequency uvulopalatoplasty as the primary treatment for OSA. Availability for follow-up sleep study data: approximately 6 months after surgery.

Exclusion Criteria: Significant multilevel pharyngeal obstruction on DISE, including tongue base or epiglottic collapse requiring additional concurrent procedure. Tonsil size greater than grade 2, as large tonsils would necessitate tonsillectomy as the primary intervention. Body mass index of over 30 to limit confounding from obesity related changes. Craniofacial abnormalities. Concurrent oropharyngeal procedures other than palatal surgery, including tonsillar cryptolysis, performed at the time of the palatal intervention, as these represent a potential confounding variable in the isolated assessment of palatal surgical outcomes. Presence of significant comorbid conditions affecting sleep, like untreated hypothyroidism, neuromuscular disease, and lack of postoperative follow-up.

### 2.3. Patient Flow

Of 124 adults identified with a confirmed OSA—mild and moderate diagnosis during the study period—28 were excluded: 13 demonstrated significant multilevel pharyngeal obstruction on DISE, 6 exhibited nasal obstructions as determined by rhinomanometry, 5 had BMI > 30 kg/m^2^, and 4 had tonsil size greater than grade 2. It should be noted that patients presenting with obvious clinical indicators of these exclusion criteria—including severe OSA, markedly elevated BMI, craniofacial/skeletal malformation, or large tonsils—were redirected to appropriate alternative management pathways at the initial triage; therefore, 124 patients identified represented those who proceeded to formal eligibility assessment. Of the remaining 96 eligible patients, 5 were lost to follow-up, and 5 had undergone concurrent oropharyngeal procedures at the time of palatal surgery, yielding a final analytic cohort of 86 patients.

The choice of procedure was not randomized. During the 2-year period, both AP and RF-UPP were routinely available in our center and selection was based on a combination of anatomic findings, patient preference, and surgeon assessment. Patients with thin or minimally redundant soft palates or strong preference for a minimally invasive option were generally steered toward RF-UPP. Conversely, patients with palatal redundancy, anteroposterior airway collapse on examination, or greater functional obstruction at the soft palate were considered better candidates for AP. All baseline demographic and clinical characteristics of the two groups were abstracted from the medical records.

### 2.4. Surgical Technique

**Figure 1:**
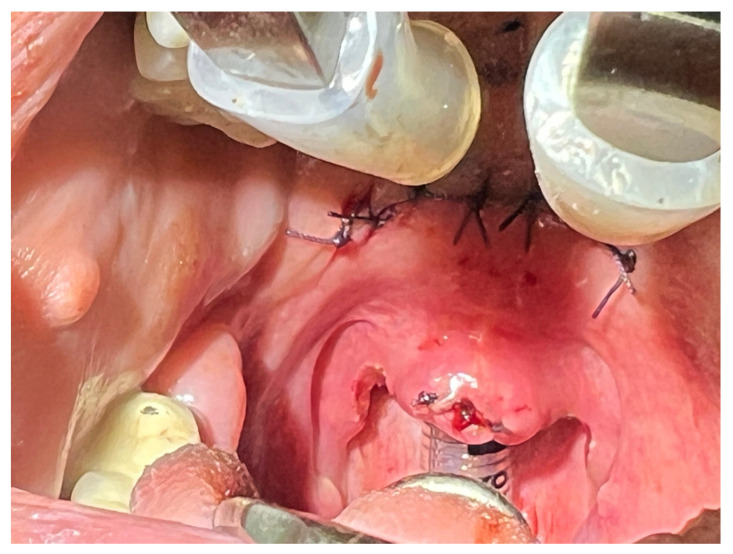
Intraoral intraoperative view during anterior palatoplasty demonstrating excision of a horizontal strip of soft palate and uvula reduction.

All surgeries were performed by 2 surgeons, using general anesthesia for the AP and local anesthesia for RF-UPP, with standardized approach to each technique. In the AP group, the anterior palatoplasty was done as described by Pang and colleagues—a horizontal strip of mucosa was excised from the soft palate, just inferior to the junction of the hard and soft palate [3]. The uvula was preserved or only partially trimmed if excessively long. The palatal musculature was then advanced anteriorly and superiorly. The edges of the mucosal incision were brought together with 2–3 absorbable sutures, effectively shortening and stiffening of the soft palate. This AP advancement and tightening increase the retropalatal airway space. Care was taken to avoid excessive resection that could have caused velopharyngeal insufficiency. The goal was to create a fibrous scar band in the soft palate for stabilization (Figure 1).

In the RF-UPP group, a radiofrequency uvulopalatoplasty was performed. This entailed submucosal radiofrequency ablation of the soft palate and uvula using a bipolar radiofrequency probe, which was delivered at 4–6 points, targeting the soft palate and the uvular base. All RF treatments were done in a single session under direct visualization.

**Figure 2:**
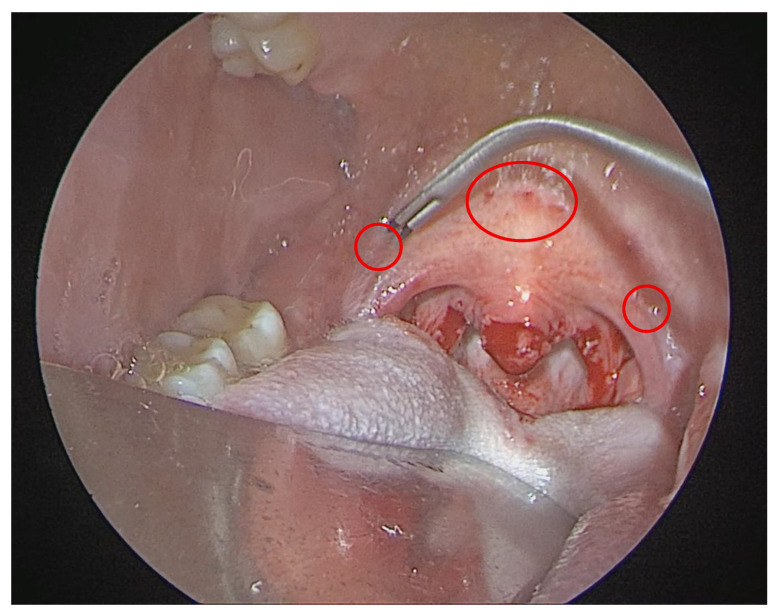
Intraoral intraoperative view during RF-UPP demonstrating the predefined target points for submucosal radiofrequency ablation and uvular volume reduction.

The mechanism of the RF-UPP is to induce thermal injury, leading to palatal tissue volume reduction and scarring over 4–6 week healing period. All patients in both groups received the same postoperative care pathway, including overnight hospital observation, analgesics, and antibiotics per standard protocol (Figure 2).

It should be noted that AP and RF-UPP represent two of several available palatal surgical techniques for OSA. Alternative approaches differ in invasiveness, underlying anatomical mechanism and reported efficacy. AP and RF-UPP were selected for this comparison because they represent two distinct points along the invasiveness–efficacy spectrum for the management of isolated anteroposterior retropalatal obstruction.

### 2.5. Outcomes and Follow-Up

The patients were evaluated at approximately 6 months post-surgery using a follow-up sleep study and questionnaires. The primary endpoint was the change in the AHI from baseline to 6 months, as measured by overnight polysomnography (PSG) in the sleep laboratory. All PSG results were scored using standard AASM criteria [25].

Secondary endpoint included changes in the Epworth sleepiness scale (ESS), an 8-item questionnaire scored 0–24, which is used to assess daytime sleepiness. Snoring severity was assessed using a 0–10 visual analog scale (VAS) (0—no snoring, 10—the worst imaginable snoring).

Sleep Specific Quality of Life—It is measured with functional outcomes of sleep questionnaire (FOSQ), an instrument where higher scores indicate a better functional status. Surgical Success Rate: It is defined as the percentage of patients achieving at least 50% reduction in the AHI from baseline and the postop AHI < 15 (for those with moderate OSA baseline)—a standard composite success definition in sleep surgery. We also noted the “cure” rate postop (AHI < 5, ESS < 10) for each group. Postoperative Pain: It is assessed through patient-reported VAS pain scores recorded in a diary for 10 days following surgery. We analyzed the peak pain score and the number of days until pain subsided to mild VAS ≤ 3. Recovery Time: It is defined as the number of days until the patient could resume normal diet and ordinary daily activities. The patients were routinely followed up at the clinic at 1–2 weeks and 6 weeks, where they reported their recovery status. Intraoperative and Postoperative Complications: They specifically focused on bleeding. Postoperative hemorrhage was defined as any episode of oropharyngeal bleeding after extubating that required medical evaluation. Other complications, infections, nasopharyngeal stenosis, voice changes, and velopharyngeal insufficiency were also noted, if present.

In summary, the preoperative diagnosis workup comprised: clinical ENT evaluation, flexible fibreoptic nasopharyngolaryngoscopy, active anterior rhinomanometry, DISE, PSG, the completion of ESS, snoring VAS, and FOSQs. The postoperative assessment at 6 months included repeat overnight PSG, the completion of ESS, snoring VAS, FOSQs, and documentation regarding postoperative pain diary, recovery milestones, and any complications.

## 3. Results

Continuous variables were summarized as mean ± SD or median (IQR). Group comparisons used Student’s *t*-test, chi-square tests, or Fisher’s exact tests, as appropriate. The AHI change was analyzed with *t*-test and ANCOVA (adjusting for baseline AHI, BMI, age, and sex). A two-tailed *p*-value < 0.05 was considered significant.

Patient Characteristics

A total of 86 patients were included, with 43 assigned to each treatment group (AP and RF-UPP). The baseline demographic and clinical characteristics were well-matched between cohorts, as detailed in Table 2.

The mean age was 42.5 ± 9.8 years in the AP group and 43.1 ± 10.5 years in the RF-UPP group. Both groups had similar body mass index (BMI ~27 kg/m^2^) and severity of obstructive sleep apnea, with mean apnea–hypopnea indices (AHI) of 22.0 ± 7.1 and 21.2 ± 6.5 events/h, respectively. The distribution of mild versus moderate OSA was also comparable between groups.

Preoperative symptom scores demonstrated no significant differences, with similar baseline Epworth sleepiness scale (ESS), snoring visual analog scale (VAS) and functional outcomes of sleep questionnaire (FOSQ) scores. Tonsil size and history of prior nasal surgery distribution was comparable between the two groups. No significant differences in comorbidities were observed.

### 3.1. Primary Outcome: AHI Reduction

At the 6-month follow-up, both procedures significantly reduced the apnea–hypopnea index (AHI) from baseline (*p* < 0.001 for both). However, AP resulted in a significantly greater reduction compared to RF-UPP, as detailed in Table 3.

The mean reduction in the AHI was −13.5 ± 7.5 events/h in the AP group, compared to −8.0 ± 7.2 events/h in the RF-UPP group (mean difference: −5.7 events/h; 95% Cl: −9.5 to −1.9; *p* = 0.004). This corresponded to a 61% improvement from baseline with AP versus 37% with RF-UPP. Consequently, the postoperative AHI was significantly lower in the AP group (8.5 ± 6.0 vs. 13.2 ± 6.5, *p* = 0.002).

The clinical superiority of AP was further demonstrated by a higher surgical success rate (79.1% vs. 53.5%, *p* = 0.012). An analysis of covariance (ANCOVA) adjusting from baseline AHI, BMI, age, and sex confirmed that the treatment group was a significant independent predictor of the 6-month AHI.

### 3.2. Secondary Outcomes: Symptom Scores and Quality of Life

Significant improvements in all patient-reported outcomes were observed at 6 months, with AP demonstrating greater efficacy than RF-UPP. The reduction in daytime sleepiness, measured by the Epworth sleepiness scale, was greater in the AP group (−5.5 ± 3.4) than in the RF-UPP group (−3.1 ± 3.2; *p* = 0.02). This resulted in a higher proportion of AP patients achieving a normal ESS score (<10) at follow-up (81.4% vs. 67.4%).

Snoring severity (VAS 0–10) was also reduced to a greater degree in the AP group (−5.7 ± 2.1) compared to the RF-UPP group (−3.1 ± 2.5; *p* = 0.002).

Sleep-specific quality of life (FOSQ) improved more substantially with AP, showing a mean gain of +2.0 ± 1.0 points versus +1.2 ± 1.1 points achieved with RF-UPP (*p* = 0.001). The overall satisfaction was high in both cohorts, with a non-significant trend favoring AP (88% vs. 76% satisfied, *p* = 0.14).

Postoperative pain and recovery

Anterior palatoplasty was associated with significantly greater postoperative morbidity and a longer recovery period than radiofrequency uvulopalatoplasty (RF-UPP). The patients in the AP group reported higher peak pain on postoperative day 1 (VAS: 6.5 ± 1.3 vs. 3.2 ± 1.1, *p* < 0.001) and a longer mean duration of significant pain (VAS > 3): 7 days (IQR 5–8) vs. on day 2 (IQR 1–3), *p* < 0.001.

Recovery milestones were also achieved sooner following RF-UPP. The median time to resume a normal diet was 2 days compared to 7 days after AP (*p* < 0.001). Similarly, the median time to return to work or to normal activities was 3 days for the RF-UPP patients versus 10 days for the AP patients (*p* < 0.001).

Safety and adverse events

No major adverse events or life-threatening complications occurred in either group. All surgeries were completed on an overnight stay basis without unplanned ICU transfers. We specifically examined bleeding complications, as palatal surgeries can pose a risk hemorrhage.

The safety profile differed between procedures, with anterior palatoplasty (AP) being associated with a higher incidence of minor bleeding events.

Intraoperative blood loss was low in both procedures. No major hemorrhages or airway complications occurred.

Other complications were infrequent and minor. Two AP patients and one RF-UPP patient experienced significant uvular edema, which resolved with medical management. No cases of permanent velopharyngeal insufficiency, significant scarring, or other serious adverse events were observed in either cohort.

In summary, at 6 months post-surgery, AP provided greater objective and subjective improvements than RF-UPP at the expense of higher postoperative morbidity. Both treatments significantly benefited patients, but AP’s efficacy edge may be relevant for patients maximal OSA resolution, while RF-UPP comfort edge may appeal to those prioritizing a quick recovery.

## 4. Discussion

In this study comparing two palatal surgical techniques for the treatment of mild and moderate OSA, anterior palatoplasty demonstrated superior objective and subjective efficacy compared to RF-UPP. The AP group achieved a significantly greater reduction in the apnea–hypopnea index (AHI) (61% vs. 37%), alongside a more substantial reduction in the daytime sleepiness and snoring intensity. These findings suggest that palatal advancement techniques like AP provide a more robust enlargement of the retropalatal airway compared to RF-UPP, which relies on localized tissues stiffening. The most pronounced difference was postoperative pain, confirming that AP’s superior efficacy comes at the cost of substantially greater early morbidity. These findings are consistent with the mechanistic distinction between the two procedures: AP structurally enlarges and stiffens the retropalatal airway through tissue advancement and fibrotic scarring, whereas RF-UPP induces localized submucosal fibrosis without anatomical repositioning.

Our findings are consistent with the established hierarchy of palatal procedures. The 79.1% success rate observed with AP aligns with the upper range of reported outcomes (57–86%) for similar advancement techniques, underscoring its reproductible efficacy in well-selected patients [13,35]. Conversely, the 53.5% success rate for a single-session RF-UPP falls within the known, more variable efficacy profile of RF ablation (25–60%) [36,37]. While RF-UPP offers a meaningful symptomatic improvement, our data objectively confirms that it is less effective than AP in terms of normalizing respiratory parameters, often leaving patients with residual mild OSA (average postop AHI (13.2 ± 6.5 vs. 8.5 ± 6.0)).

Ugur et al. reported 57.1% success at 24 months in a series of 42 mild-to-moderate patients [35]. Our 79.1% rate exceeds the most published benchmarks, likely reflecting the benefit of strict preoperative screening—specifically the use of DISE-confirmed isolated retropalatal collapse and rhinomanometry-based exclusion of nasal obstruction—which concentrated surgical candidacy in an anatomically optimal subgroup.

The 53.5% success rate for single-session RF-UPP observed in the present study is consistent with the known and variable efficacy profile of radiofrequency palatal ablation. Woodson et al.’s multi-institutional study reported a mean AHI reduction from 40.3 to 26.4 events/h at 10 weeks, with only 36.4% of patients achieving normalization (AHI < 10) [21]. Neruntarat and Chantapant reported a more favorable short-term outcome—64.9% AHI reduction at 3 months—but this has fallen to 52.8% by 14.2 months, with partial recurrence being significantly associated with weight gain [37].

Of greater clinical concern is the long-term trajectory of RF palate outcomes. Hultcrantz et al. demonstrated that despite significant snoring reduction at 6 months, only 25% of patients remained satisfied at 3 to 4 years, with additional 25% having sought additional surgical treatment [36]. These durability data suggest that while single-session RF-UPP provides meaningful short-term benefit in appropriately selected patients with mild-to-moderate OSA, it should be presented to the patient as a temporizing rather than definitive procedure [38].

AP and RF-UPP represent two points on a spectrum of palatal surgical options that vary in invasiveness, anatomical mechanisms, and reported efficacy. Understanding where these procedures sit relative to one another and to other available techniques is essential for informed surgical decision making. At the most invasive end, uvulopalatopharyngoplasty (UPPP), the historical gold standard for palatal OSA surgery, achieved a pooled success rate of approximately 40–41% in unselected population, rising to 80.6% when the selection was tightened to conform palatal-predominant collapse. UPPP carries well-documented risks, including velopharyngeal insufficiency, nasopharyngeal stenosis, and significant postoperative morbidity, which have driven the development of less respective alternative [39,40].

The magnitude of subjective improvement observed in both groups supports the clinical relevance of the findings. The mean ESS reduction of −5.5 points in the AP group and of −3.1 points in the RF-UPP group both surpassed the minimal clinically important difference (MCID) of 2–3 points for ESS in OSA [41]. The mean FOSQ improvement of +2.0 points with AP exceeded the established MCID of 1.8 points for FOSQ-10 in OSA [42], whereas the +1.2-point gain with RF-UPP improvement falls below this threshold, suggesting that the functional sleep-related quality-of-life improvement in RF-UPP, while statistically significant, may not reach clinical perceptibility for an average patient.

Positioning the present findings within the broader landscape of palatal surgical techniques, the 79.1% success rate observed with AP in this DISE-selected cohort is comparable to the upper range reported for expansion sphincter pharyngoplasty (ESP—64.8–90%) and barbed reposition pharyngoplasty (65.4–93%) in recent systematic reviews [43,44]; however, a direct comparison is limited by differences in patient selection, collapse type, and outcome definitions. ESP and BRP are primarily indicated for lateral pharyngeal collapse, whereas AP targets anteroposterior velar obstruction—the predominant pattern in the present cohort. This anatomical specificity underscores that procedure selection should be guided by the DISE-confirmed collapse pattern rather than by ranking techniques against one another. The trend away from UPPP—which declined from 25.6% of palatal procedures in 2001–2010 to 12.6% in 2011–2018 [45]—reflects the fields’ shift toward less ablative, anatomically targeted reconstructive techniques such as AP, ESP, and BRP.

The outcomes achieved in this study cannot be interpreted without reference to the preoperative selection protocol applied, as all patients underwent active anterior rhinomanometry and DISE, and only those with isolated or predominant retropalatal collapse were included.

The present study focuses specifically on palatal surgical techniques; however, it is essential to contextualize these findings within the full spectrum of OSA management, in which surgical intervention represents only one pathway within a broader treatment algorithm. CPAP therapy remains first-line and most effective treatment for moderate and severe OSA, with well-established effects on the AHI normalization and cardiovascular risk reduction. However, long-term CPAP adherence is a persistent clinical challenge, particularly in mild-to-moderate disease—the precise population represented here. Oral appliance therapy provides a validated non-surgical alternative for mild-to-moderate OSA and CPAP intolerant patients, with AASM endorsing the use as a first-line option in this severity range. When anatomy, patient preference, or CPAP failure drives consideration of surgical intervention, the choice of procedure must be guided by the site and pattern of pharyngeal collapse rather than AHI severity alone [2,21].

Palatal surgery is appropriately indicated when the primary obstruction site is the velum, as confirmed by DISE. In patients with significant tongue base collapse, epiglottic obstruction, or multilevel disease, palatal surgery alone is insufficient and multilevel approaches incorporating tongue base procedures, maxillomandibular advancement, or hypoglossal nerve stimulation are required.

Clinical Interpretation and Implications

The efficacy of both procedures is contingent on appropriate patient selection. Our findings are derived from a cohort with retropalatal obstruction and with a BMI < 30, which represents the ideal anatomical candidate for palatal surgery. In this population, AP demonstrated superior outcomes across all OSA severity subgroups, suggesting that its advantages are not limited to a specific patient profile within the selected group.

The robust, parallel improvements in both objective (AHI) and subjective (ESS, QoL snoring VAS) outcomes with AP lend strong support to its clinical validity. The snoring reduction achieved by AP is likely based on the biomechanical action of anteriorly advancing the soft palate, which may provide a more definitive structural modification than the stiffening induced by RF-UPP. For patients with more severe baseline OSA (AHI ≥ 20), our data suggest that the superior and potentially more durable AHI reduction achieved with AP may offer greater long-term benefits.

The clinical advantage of AP’s superior efficacy is achieved at the expense of a more strenuous postoperative recovery. Patients in the AP group experienced significantly greater pain and a longer convalescence than those treated with RF-UPP, a result that reflects the expected morbidity of an open surgical dissection versus a minimally invasive ablation. Consequently, for patients who want to minimize recovery time, RF-UPP presents a low morbidity alternative, albeit a more modest efficacy.

However, the morbidity of AP should be viewed in context. Our data, as well as the literature, suggest its profile is more favorable than that of traditional UPPP, likely due to its tissue-preserving approach. In our series, AP-related pain was moderate, manageable, and not associated with serious complications.

Several additional observations merit discussions. First, the concordance between objective (AHI) and subjective (ESS, snoring VAS, FOSQ) improvements was robust in both groups, with all outcome measures moving in the same direction and reaching statistical significance. This parallel improvement strengthens the validity of both the objective and subjective assessment. Second, despite the absence of randomization, the two groups were well-matched across all baseline parameters, with no statistically significant difference between them. Third, the absence of serious complications in either group, as velopharyngeal insufficiency, nasopharyngeal stenosis, or significant hemorrhage is consistent with the established safety profile of both techniques and supports their suitability for routine clinical practice in appropriately selected patients.

For surgeons, these findings clarify the role of the two distinct palatal procedures. In patients with mild-to-moderate OSA and retropalatal collapse, AP may be presented as the highest-efficacy option, offering a high likelihood of major AHI reduction and snoring resolution. RF-UPP should be offered as the low-morbidity alternative for patients who prioritize minimal recovery disruption, accepting that the AHI may remain in the mild range and repeated procedures may be necessary.

Patient preference is the deciding factor. Some patients will opt for more definitive, one-stage solution offered by AP despite the 1–2 week recovery. Others will choose the gentler recovery offered by RF-UPP.

Practically, the one-time nature of AP may prove more cost-effective than multiple RF-UPP sessions. Furthermore, AP typically meets insurance criteria for OSA treatment, while RF-UPP is sometimes categorized as a snoring-eliminating procedure. Both techniques demonstrated excellent safety, confirming AP as a less morbid evolution of UPPP and RF-UPP as a low-risk, first-line intervention.

Study limitations

Several limitations should be considered. First, the retrospective, non-randomized design introduces potential for selection bias, despite the comparability of baseline characteristics. Unmeasured confounders, such as subtle anatomical differences, may have influenced outcomes. Second, the 6-month follow-up, while adequate for the initial assessment precludes the evaluation of long-term durability, although the existing literature suggests that the AP results remain stable [4]. Third, the absence of routine cephalometry or CBCT imaging represent a limitation, as subtle skeletal contributions to retropalatal collapse cannot be entirely excluded. While gross dentofacial deformity was not clinically evident in this cohort, undetected skeletal contributions remain a potential confounder, and further studies should incorporate standardized imaging to allow the characterization of the bony airway anatomy.

Secondary PSG parameters beyond the AHI—including minimum SpO_2_, oxygen desaturation index (ODI), arousal index, and sleep architecture—were not systematically collected in this study. This represents a limitation, as these parameters may provide additional insight into the physiological impact of surgical intervention. However, this limitation is consistent with much of the existing literature on palatal surgery. For example, Pang et al.’s systematic review of anterior palatoplasty found that only a minority of studies reported minimum SpO_2_, with no study reporting on the arousal index or sleep architecture data [4]. Similar data on these parameters remains limited for RF-UPP, with most available evidence focusing primarily on the AHI. To date, only a small number of studies had explored changes in sleep architecture pharyngoplasty technique, with non-significant findings in the preliminary stage [46]. Future prospective studies incorporating comprehensive polysomnographic analysis are warranted to better characterize the full physiological impact of sleep surgery.

Finally, the lack of blinding could have biased patient-reported outcomes, though the concordance between subjective and objective measures strengthened our findings.

Our conclusions are directly applicable to patients with mild-to-moderate OSA and isolated retropalatal obstruction. The results should not be extrapolated to individuals with severe OSA, significant multilevel collapse, or higher BMI, who typically require more extensive intervention. Furthermore, the technical proficiency required for AP and the specific RF technology used may influence outcomes; thus, our results reflect the experience of a specialized, experienced surgical practice.

## 5. Conclusions

The study provides a comprehensive comparison of AP versus RF-UPP for mild-to-moderate OSA. Anterior palatoplasty was associated with greater improvement in objective respiratory parameters and key symptoms, including daytime sleepiness and snoring, compared to RF-UPP. However, the superior efficacy was achieved at the expense of greater postoperative pain and a longer recovery time, whereas RF-UPP offered minimal discomfort and a rapid return to normal activities, albeit more modest clinical improvements.

In balancing these findings, we conclude that AP may offer an effective surgical option, particularly for patients who prioritize maximal symptom relief and who are willing to accept a short-term increase in recovery burden.

RF-UPP remains a valuable minimally invasive treatment with very low morbidity, suitable for patients who prioritize a quick recovery or who have contraindications to more invasive surgery. The choice between procedures should be individualized based on patient anatomy, OSA severity, and treatment goals.

Our results contribute to the growing evidence supporting palatal advancement techniques and suggest that AP yields comparable outcomes to or better than traditional palatal surgeries. Future prospective trials should confirm long-term durability and investigate combined approaches.

## Figures and Tables

**Table 2:** Baseline characteristic of patients undergoing AP vs. RF-UPP.

Characteristic	AP Group (*n* = 43)	RF-UPP Group (*n* = 43)	*p*-Value
Age, years	42.5 ± 9.8	43.1 ± 10.5	0.78
Male sex, %	30 (69.8%)	29 (67.4%)	0.82
Body mass index, kg/m^2^	27.2 ± 2.9	26.5 ± 3.1	0.34
AHI (events/h)—baseline	22.0 ± 7.1	21.2 ± 6.5	0.60
- Mild OSA	17 (39.5%)	19 (44.2%)	0.67
- Moderate OSA	26 (60.5%)	24 (55.8%)	0.67
ESS score (0–24)—baseline	11.0 ± 3.8	10.3 ± 4.0	0.42
Snoring VAS (0–10)—baseline	8.4 ± 1.5	8.2 ± 1.6	0.57
FOSQ score (5–20)—baseline	16.0 ± 2.5	16.3 ± 2.8	0.64

Data are mean ± SD or percentage of patients. There were no statistically significant differences between the AP and RF-UPP groups in any baseline parameter (all *p* > 0.05). AHI—apnea–hypopnea index; OSA—obstructive sleep apnea, ESS—Epworth sleepiness scale; VAS—visual analog scale; FOSQ—functional outcomes of sleep questionnaire.

**Table 3:** Key 6—month outcomes and morbidity in AP vs. RF-UPP groups.

Outcome (6 Months)	AP (*n* = 43)	RF-UPP (*n* = 43)	Between-Group *p*
Average AHI, events/h	8.5 ± 6.0	13.2 ± 6.5	0.002
-% AHI reduction	61%	37%	-
Success rate (AHI improvement ≥ 50% and <15)	34/43 (79.1%)	23/43 (53.5%)	0.012
Mean AHI reduction	−13.5 ± 7.5	−8.0 ± 7.2	<0.001
ESS score (0–24)	6.0 ± 2.8	7.3 ± 3.1	0.001
ESS ≤ 10 (% patients)	35/43 (81.4%)	29/43 (67.4%)	0.13
mean reduction	−5.5	−3.1	0.001
Snoring VAS (0–10)	2.6 ± 2.1	4.0 ± 2.5	0.002
Snoring resolved (VAS ≤ 3, %)	38/43 (88.4%)	31/43 (72.1%)	0.048
Mean reduction	−5.7	−3.1	0.002
FOSQ score (5–20)	+2.0 ± 1.0	+1.2 ± 1.1	0.001
Postop peak pain VAS (0–10)	6.5 ± 1.3	3.2 ± 1.1	<0.001
Days with pain > 3 (median)	7 (IQR 5–8)	2 (IQR 1–3)	<0.001
Return to normal diet, days	7 (IQR 6–10)	2 (IQR 1–3)	<0.001
Return to work/usual activity, days	10 (IQR 8–12)	3 (IQR 2–4)	<0.001
Any serious complication	0	0	-

Values are mean ± SD or percentage of patients. Between-group comparison of mean change (AP VS RF-UPP). Fisher’s exact test used for proportions. AHI: apnea–hypopnea index; ESS: Epworth sleepiness scale; VAS: visual analog scale; FOSQ: functional outcomes of sleep questionnaire.

## Data Availability

The data supporting the findings of this study are available from the authors upon reasonable request. The data are not publicly available due to institutional data protection and patient privacy restrictions.

## Abbreviations

The following abbreviations are used in this manuscript:

AP	anterior palatoplasty
RF-UPP	radiofrequency-assisted uvulopalatoplasty
OSA	obstructive sleep apnea
AHI	apnea–hypopnea index
BMI	body mass index
ESS	Epworth sleepiness scale
VAS	visual analog scale
FOSQ	functional outcomes of sleep questionnaire
PSG	polysomnographic
UPPP	uvulopalatopharyngoplasty
